# Stress cardiac MRI provides excellent prognostic risk stratification in coronary artery disease: a systematic review of the literature

**DOI:** 10.1186/1532-429X-15-S1-E38

**Published:** 2013-01-30

**Authors:** Michael J Lipinski, Courtney M McVey, Jeffrey S Berger, Christopher M Kramer, Michael Salerno

**Affiliations:** 1 Medicine, University of Virginia, Charlottesville, VA, USA; 2 University of Virginia, Charlottesville, VA, USA; 3 Radiology, University of Virginia, Charlottesville, VA, USA; 4 Medicine, New York University, New York, NY, USA

## Background

While the diagnostic accuracy of stress cardiac magnetic resonance imaging (CMR) for detecting obstructive coronary artery disease (CAD) has been established, the prognostic value of stress CMR is less well described in the literature. Thus, we performed meta-analysis to study the role of stress CMR in assessing cardiovascular prognosis.

## Methods

CENTRAL, mRCT, and PubMed were searched for eligible studies that provided greater than 6 months of prognostic data on patients that underwent stress CMR. The primary end-points evaluated were cardiovascular mortality, myocardial infarction, and the combined endpoint of cardiovascular mortality or myocardial infarction. Pooling was performed using a random-effect model with summary effect estimates (95% confidence intervals) and annualized event rates were assessed. Values presented as mean ± standard error of the mean.

## Results

**Figure 1:**
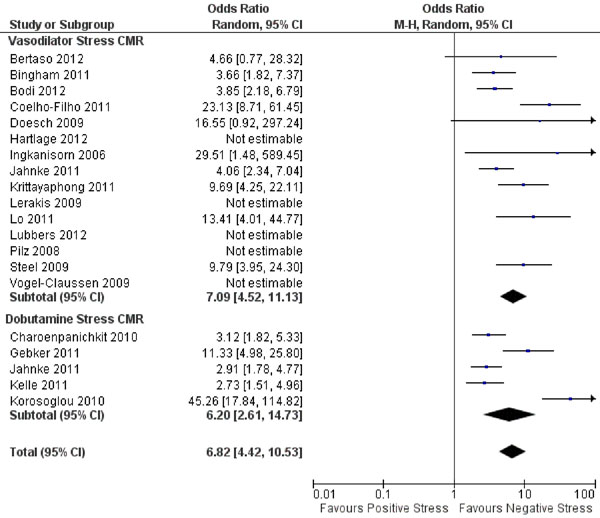


**Figure 2:**
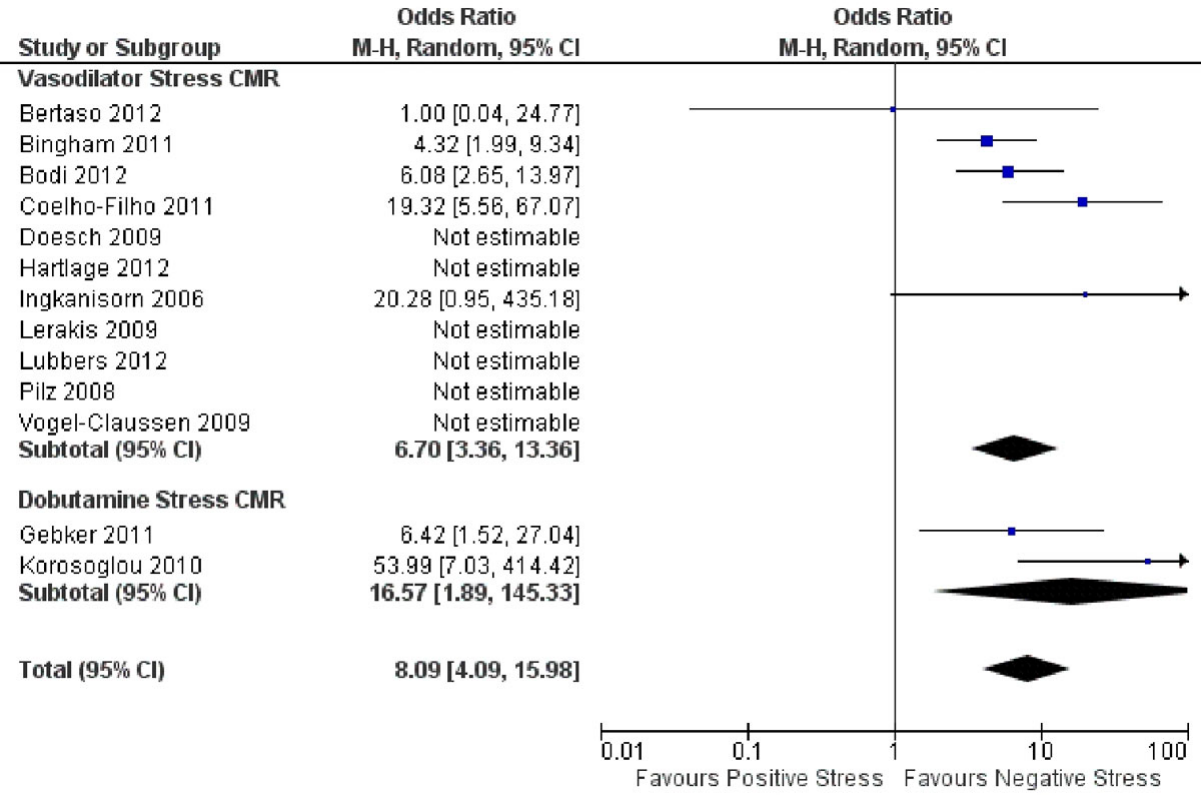


Data was included from 19 studies (13 vasodilator, 4 dobutamine, and 1 that used both) with a total of 10,573 patients and an average follow-up of 27 months. Patients had a mean age of 61 years, 58% were male, 19% had a prior MI, 65% had hypertension, 58% had hyperlipidemia, and 24% had diabetes mellitus. CMR demonstrated a mean LV ejection fraction of 60% and stress testing demonstrated ischemia in 27% of patients. Studies demonstrated that patients with positive stress CMR had significantly increased combined outcome (Figure 1), cardiovascular death (Figure 2), and myocardial infarction compared with negative stress CMR. The combined outcome annualized events rates were 5.3% for positive stress tests versus 0.8% for negative stress tests (p=0.0002), 2.0% versus 0.2% for cardiovascular death (p=0.02), and 2.6% versus 0.2% for myocardial infarction (p=0.003). When comparing vasodilator with dobutamine stress CMR, there were no significant differences between combined cardiovascular outcomes for positive (5.4% versus 4.8%, respectively) or negative studies (0.7% versus 1.0%, respectively).

## Conclusions

A negative stress CMR study (vasodilator or dobutamine) is associated with very low risk of cardiovascular mortality or myocardial infarction. Thus, stress CMR has excellent prognostic characteristics comparable to stress echocardiography or stress nuclear imaging and may help guide risk stratification of patients presenting with known or suspected CAD.

## Funding

AHA 10SDG2650038, NIH K23 HL112910-01

